# Functional evidence on the involvement of the *MADS-box* gene *MdDAM4* in bud dormancy regulation in apple

**DOI:** 10.3389/fpls.2024.1433865

**Published:** 2024-07-15

**Authors:** Janne Lempe, Mirko Moser, Elisa Asquini, Azeddine Si-Ammour, Henryk Flachowsky

**Affiliations:** ^1^ Julius Kühn Institute (JKI), Federal Research Centre for Cultivated Plants, Institute for Breeding Research on Fruit Crops, Dresden, Germany; ^2^ Research and Innovation Centre, Fondazione Edmund Mach (FEM), San Michele all’Adige, TN, Italy

**Keywords:** winter dormancy, DAM gene, *Malus* × *domestica*, growth cessation, climate change, transgenic apple

## Abstract

Over the course of the year, temperate trees experience extremes in temperature and day length. In order to protect themselves from frost damage in winter, they enter a dormant state with no visible growth where all leaves are shed and buds are dormant. Also the young floral tissues need to withstand harsh winter conditions, as temperature fruit trees like apple develop their flower buds in the previous year of fruit development. So far, the genetic control of induction and release of dormancy is not fully understood. However, the transcription factor family of *DORMANCY-Associated MADS-box (DAM)* genes plays a major role in the control of winter dormancy. One of these genes is *MdDAM4*. This gene is expressed in the early phase of bud dormancy, but little is known about its function. Six transgenic apple lines were produced to study the function of *MdDAM4* in apple. For plant transformation, the binary plasmid vector p9oN-35s-MdDAM4 was used that contains the coding sequence of *MdDAM4* driven by the 35S promoter. Transgenicity of the lines was proven by PCR and southern hybridization. Based on siRNA sequencing and phenotypic observations, it was concluded that line M2024 overexpresses *MdDAM4* whereas the gene is silenced in all other lines. Phenotyping of the transgenic lines provided evidence that the overexpression of *MdDAM4* leads to an earlier induction and a later release of dormancy. Silencing this gene had exactly the opposite effects and thereby led to an increased duration of the vegetation period. Expression experiments revealed genes that were either potentially repressed or activated by *MdDAM4*. Among the potentially suppressed genes were several homologs of the *cytokinin oxidase 5* (*CKX5*), five *LOX* homologs, and several expansins, which may indicate a link between *MdDAM4* and the control of leaf senescence. Among the potentially activated genes is *MdDAM1*, which is in line with observed expression patterns during winter dormancy. *MdDAM2*, which shows little expression during endodormancy also appears to be activated by *MdDAM4*. Overall, this study provides experimental evidence with transgenic apple trees for *MdDAM4* being an important regulator of the onset of bud dormancy in apple.

## 1 Introduction

In the temperate climate zone, deciduous fruit trees like the apple *Malus × domestica* Borkh. experience extreme cycles of temperature and day length. Warm summers alternate with cold and harsh winters. As an adaptive strategy, perennial tree species alternate phases of high metabolic activity, growth, and reproduction with phases of dormancy with no visible growth. The dormancy phase begins in autumn, when trees stop growing shoots and shed their leaves. During dormancy, the trees have no leaves and woody bud scales, which protect the apical shoot meristem from extreme weather conditions, surround the buds. Once dormancy is complete, the buds start to take up water and begin to swell. This is followed by bud burst, blooming, and the formation of new fruits, shoots, and leaves. The timing of phase transitions, and thereby the length of the growing season, is tightly controlled by environmental cues such as light and ambient temperature (Heide, 2008; Cooke et al., 2012). In apple, it is ambient temperature rather than photoperiod that controls the timing of dormancy (Heide and Prestrud, 2005; Takeuchi et al., 2018). Winter dormancy itself can be divided in at least three distinct phases: para-, endo-, and ecodormancy (Lang, 1987). During paradormancy, a change in growth conditions can reverse the inhibition of growth. This is not possible during endodormancy (deep dormancy), where buds are fully dormant and growth cannot be evoked by changing environmental conditions. Only after a cultivar-specific amount of cold has been perceived the transition to ecodormancy occurs. During ecodormancy, a certain amount of heat hours accumulate before bud break can occur.

Because the control of winter dormancy is highly dependent on ambient temperature, global warming strongly affects the timing of winter dormancy in several ways. First, if the amount of cold (chill) hours decreases during winter, the cold requirement during endodormancy may not be fulfilled. Second, due to warmer spring temperatures the heat requirement during ecodormancy is met earlier. If the cold requirement is not met, a delay in bud break, a loss of flower synchronization, a reduction in flower quality, and an overall reduction in yield and fruit quality have been observed (Atkinson et al., 2013; Legave et al., 2013). Temperature forecast predicts an increase in the frequency of years in which the cold requirement will not be met in cultivars with high chilling requirements (Fernandez et al., 2023), especially for the Mediterranean region as well as for southern German fruit-growing regions (Pfleiderer et al., 2019). The negative consequences of meeting the heat requirement earlier in the spring are an earlier bud break combined with earlier flowering. For many plant species, a clear trend of an ever-earlier start of bud break and flowering in spring can be observed, which is in line with global warming (Vitasse et al., 2022). The problem associated with an early bud break is an increase in the risk of damage by late spring frost events (Unterberger et al., 2018), which can lead to extremely high economic losses in individual years. Whether the cold requirement can be met and whether the risk of damage from spring frost increases depend on both specific climatic conditions and the genotype of the respective cultivar. The risk of frost damage increases particularly sharply if the time of bud break advances faster than the time of the last spring frosts, as can be observed at altitudes above 800 m in Switzerland or in southern German fruit-growing areas, for example (Pfleiderer et al., 2019; Vitasse et al., 2022).

The molecular control of the onset, phase transitions, and the release of winter dormancy is not yet fully understood. The SVP-like MADS-box transcription factor families with *MdSVPa*, *MdSVPb*, and *DORMANCY-Associated MADS box (DAM)* genes however have been found to play a key role in the control of winter dormancy in perennial species. The *DAM* gene family was first identified in the natural *P. persica* mutant *evergrowing*, originating from central Mexico (Rodriguez et al., 1994; Bielenberg et al., 2008). This mutant is non-dormant and lacks a cluster of six *DAM* genes. Subsequently, members of the *DAM* gene family have been found in rather unrelated plant species (Quesada-Traver et al., 2022) like leafy spurge (Horvath et al., 2010), kiwi (Wu et al., 2012), aspen (Singh et al., 2018), and grapevine (Vergara et al., 2021) and in several fruit tree species of the *Rosacea* plant family (Saito et al., 2013; Mimida et al., 2015; Porto et al., 2015; Wisniewski et al., 2015; Kumar et al., 2016). Surprisingly, *DAM* genes have also been found in the evergreen non-winter dormant loquat *Eriobotrya japonica*, where *DAM* activity was speculated to be associated with a summer dormant period (Quesada-Traver et al., 2022).

Particular expression patterns during winter dormancy, as well as experiments with transgenic apple trees that miss-express single or multiple *MdDAM* or *MdSVP* genes, have solidified their essential role in controlling winter dormancy (Wu et al., 2017; Moser et al., 2020). *MdDAM1* and *MdDAM4* show a peak of expression during endodormancy, whereas *MdDAM2* expression appears to peak before the start of endodormancy (Moser et al., 2020; Falavigna et al., 2021; Lempe et al., 2022). The expression pattern of *MdDAMb* is less clear and appears to differ between climatic growth conditions (Wu et al., 2017; Falavigna et al., 2021). *MdSVPa* and *MdSVPb* are broadly expressed throughout endo- and ecodormancy (Falavigna et al., 2021; Lempe et al., 2022). Both *MdSVP* proteins have been shown to form heteromeric transcription factor complexes with specific *MdDAM* proteins as well as with *MdFLC-like* (Falavigna et al., 2021). Depending on the availability of the interacting protein partners, such complexes control phase-specific sets of downstream targets (Falavigna et al., 2021).

Functional analysis of *MdDAM* and *MdSVP* genes confirmed their essential role in dormancy control. Overexpression of the Japanese apricot (*P. mume*) homolog of *DAM6* in poplar caused early growth (Sasaki et al., 2011). In apple, transgenic trees in which all *MdDAM* and *MdSVP* genes are silenced were not able to enter bud dormancy in winter (Wu et al., 2021). Silencing of *MdDAM1* only was sufficient to prevent the onset of winter dormancy and lead to an evergreen and non-dormant growth habit (Moser et al., 2020). Overexpression of *MdDAMb* and *MdSVPa* resulted in a delayed bud break and constrained lateral shoot outgrowth; however, no change in flower and fruit development was observed (Wu et al., 2017).

Duplication and diversification of MADS-box genes has been an important mechanism for the evolution of functional innovation (Kaufmann et al., 2005). Therefore, it is questionable whether *MdDAM1* is the only DAM gene that controls dormancy in apples. This question arises particularly in light of the fact that *MdDAM4*, another *DAM* gene of apple, shows an expression pattern very similar to *MdDAM1.* However, the functional role of *MdDAM4* has not yet been studied in detail. Here in this study, we focused on the functional characterization of *MdDAM4* in transgenic apple trees in which the gene was either constitutively silenced or overexpressed. Our results clearly indicate *MdDAM4*’s functional role in the control of endodormancy. With reduced levels of *MdDAM4*, trees extended their vegetative period and did not enter dormancy. Overexpression lead to a reduction of the vegetative period by early growth cessation, bud termination, and leaf fall. Genes associated with altered *MdDAM4* expression are likely involved in leaf senescence.

## 2 Materials and methods

### 2.1 Construction and validation of transgenic apple lines

**Figure 1:**
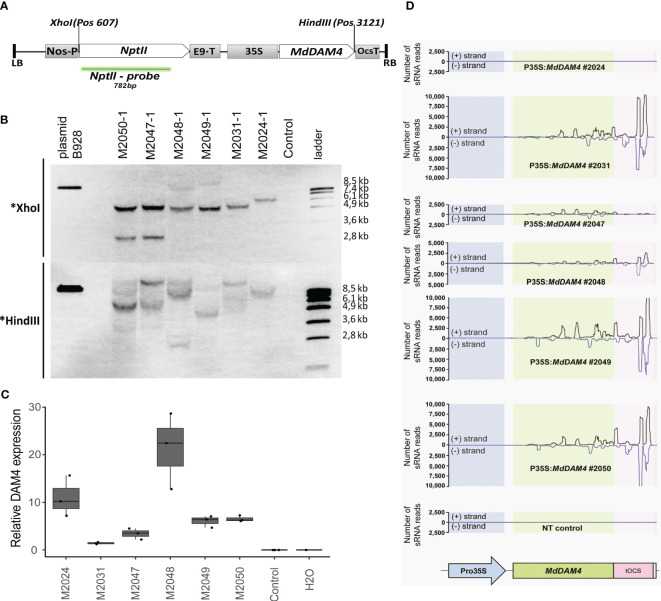
Molecular characterization of *MdDAM4* transgenic apple lines. **(A)** Schematic representation of the *MdDAM4* transgene. Between left and right borders are the *neomycin phosphotransferase II resistance gene (nptII)* under the NOS promoter and with the rbcS-E9 termination signal, the 35S promoter, the *MdDAM4* coding sequences, and the OCS terminator. Further indicated are the positions of the cutting site of the restriction enzymes XhoI and HindIII, as well as the nptII probe. **(B)** Southern blot of genomic DNA digested either with XhoI or HindIII. **(C)** qRT-PCR analysis of *MdDAM4* expression. **(D)** Analysis of siRNAs that map to the sequence of *MdDAM4* transgene.

Proliferating *in vitro* shoot cultures of the *Malus × domestica* Borkh. cultivar ‘Gala’ were transformed with the *A. tumefaciens* strain EHA105, containing the plasmid vector p9oN-35s-*MdDAM4* (GenBank accession number OR394976) using leaf disc transformation as described by Flachowsky et al. (2007). Leaf discs of ‘Gala’ not treated with *A. tumefaciens* were used as non-transformed control. Transformed tissue and non-transformed tissue of ‘Gala’ (control) were regenerated and propagated as described previously (Flachowsky et al., 2007). The plasmid vector p9oN-35s-*MdDAM4* was constructed by the DNA Cloning Service (Hamburg, Germany). The T-DNA of this vector contains the *neomycin phosphotransferase II* (*nptII*) resistance gene driven by the NOS (nopaline synthase) promoter and the rbcS-E9 termination signal of *Pisum sativum*. The reading frame of *nptII* was optimized for gene expression in plants. The T-DNA further contains the coding sequence (CDS) of the *MdDAM4* gene of the apple cultivar ‘Golden Delicious’ under the control of the *Cauliflower mosaic virus* 35S promoter and the terminator of the octopine synthase (ocs) gene (**Figure 1A**).

Non-transformed control shoots of ‘Gala’ and transgenic shoots were grown *in vitro* on M8 media. After successful propagation, shoots were micrografted onto ‘Golden Delicious’ seedlings and planted into 8-cm to 14-cm plastic pots filled with soil. After cultivation for 12 months in the greenhouse, bud wood was collected and grafted onto M9 rootstocks. Before transferring potted trees to an insect protection tent for phenotypic assessment under conditions very similar to the open field, trees were grown for 2 to 3 years in the greenhouse.

The QIAGEN DNeasy Plant Mini Kit (Qiagen, Hilden, Germany) was used for extracting genomic DNA of transgenic lines. The presence of the transgene was confirmed with the primers 35S_F and JL009, and the absence of bacterial culture from trees was confirmed with primers VirG_F and VirG_R (**Supplementary Table 1**). Primers DAMox-F and OCSterm_R were used to confirm insertion of full-length CDS of *MdDAM4* by PCR. Southern blots were performed as described in Moser et al. (2020). The genomic DNA was digested with *Xho*I or *Hin*dIII (Thermo Fisher Scientific Inc.) separately, overnight at 37°C, and DNA was hybridized with a DIG-labeled *nptII*-probe. The *nptII* probe was generated using the primers nptII_opt_F and nptII_opt_R, and the signal was detected using the anti_DIGAP (Roche Deutschland Holding GmbH) and ECF substrate (Amersham Biosciences Europe GmbH, Freiburg, Germany) on a ChemiDoc XRS+ System (Bio-Rad Laboratories GmbH). The DIG-labeled DNA Molecular Weight Marker VII (Roche, Germany) was used as ladder.

### 2.2 SiRNA analysis

For siRNA analysis, terminal buds were collected 01/12/2020 and 01/02/2021. siRNA was isolated from single buds, one per line and time point, by using the mirPremier kit from Sigma-Aldrich (Merck KGaA, Darmstadt, Germany). The TruSeq Small RNA Library Prep Kit (Illumina, Italy) was used to prepare the sRNA libraries. The libraries were sequenced on a MiSeq (Illumina) platform at the Edmund Mach Foundation (San Michele all’Adige, Italy) with the configuration 1 × 36 bp with the Miseq Reagent Kit v3 yielding around 16.5 M raw reads.

### 2.3 Expression analysis

For expression analysis, three terminal buds were collected from the lowest three shoots for each of the three replicate trees on 06/10/2020, flash frozen, and homogenized. For ‘Gala’ control trees, a mix of replicates 1 and 2 was used as third replicate, since the third tree was excluded from analysis due to disease. RNA was extracted by using the InviTrap^®^ Spin Plant RNA Mini Kit (Invitek, Berlin, Germany), and DNAse was removed from RNA samples by using the DNA-free™ kit (Thermo Fisher Scientific Inc.). The RevertAid first-strand cDNA synthesis kit (Thermo Fisher Scientific Inc.) was used with oligo_(dT)_ primer to synthesize cDNA. qRT-PCR was performed with the Maxima qPCR Master Mix (Thermo Fisher Scientific Inc.) on a CFX96 Touch Real-Time PCR detection system (Bio-Rad Laboratories, Inc., Hercules, CA, U.S.A.). The primers DAM4ox_F and DAM4ox_R were used to measure the relative expression of the *MdDAM4* transgene (**Supplementary Table 1**).

### 2.4 RNA-seq analysis

For RNA-seq experiments, RNA samples collected on 06/10/2020 (see above) of the lines M2024 and M2031 and control plants were selected. For each RNA sample, an average of 30.9 Mio reads was generated for each replicate by the Novogene UK Cambridge Sequencing Center.

The RNA-seq raw reads were cleaned using Trimmomatic with standard parameters (Bolger 2014). Cleaned reads were aligned an unpublished ‘Gala’ genome, as well as to the reference transcriptome dataset of Malus × domestica (ASM211411v1) downloaded from NCBI (GCA_002114115.1, integrated with ESTs manually downloaded) by using Bowtie2 with standard parameters. Abundances of reads unique for each transcript were counted for each replicate for the overexpressing, the silenced, and the non-transgenic ‘Gala’ control plants and reported in a raw abundance matrix. The count matrix was then subjected to differential expression analysis using the DESeq2 R module performing a Maximum Likelihood test to calculate the fold change against the non-transformed control with default parameters (Love et al., 2014). Transcripts with an adjusted p-value <0.05 and an absolute value for the log2 fold change (log2FC) ≥1 were considered as differentially expressed. The differentially expressed cDNAs were blasted against the GDDH13 v1.1 proteome, and the annotation of this genome was used for functional interpretations. Furthermore, we focused also on the subset of transcripts passing the filter adjusted p-value <0.01. For each transcript, the log2FC values from the pairwise comparison against the ‘Gala’ control were retrieved from the DESeq2 calculations.

### 2.5 Gene ontology analysis

The gene ontology enrichment analysis was performed using the GO Analysis Toolkit and Database for Agricultural Community AgriGO v2.0 (http://systemsbiology.cau.edu.cn/agriGOv2/) providing the ID of the differentially expressed genes to the toolkit submission form under the Rosaceae group analysis using the GDDH13 v 1.1 homology with Arabidopsis as suggested background (Tian et al., 2017).

### 2.6 Plant phenotyping and growth conditions

For phenotypic assessment, transgenic and non-transgenic control trees were grown in an insect protection tent under natural conditions without artificial lighting or temperature regulation, year-round. Trees were pruned to the same height in spring. Length measurements of the annual shoot growth as well as total tree height was determined in fall 2021 after leaf fall. Phenotyping for the date of side shoot and main shoot termination was determined weekly from 13/07/2020 to 20/11/2020. Statistical analysis and graphs were done with R studio and the packages ggplot2 and ggpubr (Wickham, 2016; Kassambara, 2023).

## 3 Results

### 3.1 Molecular characterization of the transgenic apple trees containing the 35S:*MdDAM4* transgene

To functionally characterize *MdDAM4*, transgenic ‘Gala’ apple trees were developed using *A. tumefaciens*-mediated leaf disc transformation using a transgenic cassette containing the coding sequence of the *MdDAM4* gene under the control of the 35S promoter (**Figure 1A**, GenBank accession OR394976). Six putative transgenic lines were selected after transformation on a regeneration medium containing kanamycin as a selection agent. The presence of the transgenic sequences in these lines was confirmed by PCR using the primers 35S_F and DAM4ox_R (**Supplementary Figure 1**). To determine the number of transgene insertions per line, southern blot analyses were performed by using a probe against the kanamycin resistance gene *nptII*. Genomic DNA was cut either with the restriction enzyme *Xho*I or with *Hin*dIII (**Figure 1B**). The Southern blots of all lines suggest a single insertion event for line M2024, and multiple T-DNA insertions for lines M2031-M2050 (**Figure 1B**). Since the hybridization patterns are different for all lines (**Figure 1B**) and the lines originate from different explants, it can be concluded that the lines represent six individual transformation events.

A qRT-PCR approach was carried out to examine the expression status of the *MdDAM4*-containing transgenic cassette (**Figure 1C**). The expression of the transgenic *MdDAM4* copy was very high in lines M2024 and in line M2048 and showed intermediate expression levels in lines M2049 and M2050. No expression was found in the non-transgenic control, as expected.

To investigate whether the transgenic lines overexpress the *MdDAM4* gene or whether the transgenic expression leads to silencing of the endogenous *MdDAM4* gene, the occurrence of siRNA molecules with sequence identity to *MdDAM4* was tested. No siRNAs mapped to *MdDAM4* in the non-transgenic control as expected, as well as in line M2024. The absence of siRNAs that mapped to *MdDAM4* is consistent with the overexpression of the transgene (**Figure 1D**). In contrast, a large number of siRNAs mapped to *MdDAM4* and the OCS terminator in lines M2031, M2049, and M2050. *MdDAM4*-siRNAs were also present in lines M2047 and M2048, although the number was lower than in lines M2031, M2049, and M2050. Based on these results, it was concluded that line M2024 appears to overexpress *MdDAM4*, whereas in lines M2031, M2047, M2048, M2049, and M2050, the gene seems to be silenced.

### 3.2 Transgenic *MdDAM4* lines show altered timing of growth and dormancy

**Figure 2:**
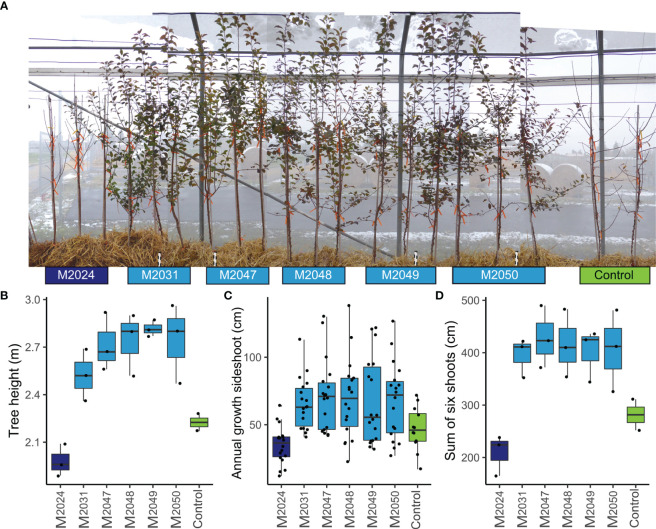
Transgenic apple trees, which differ in *MdDAM4* transcription levels, show altered behavior with respect to growth cessation and annual growth compared with control. **(A)** photograph of control and transgenic trees with three replicate trees per line, growing inside an insect protection tent, taken on 01/12/2020. **(B)** Tree height was measured from soil level to the highest terminal bud. **(C)** The annual growth of each side shoot was determined by measuring wood length from tip to the beginning of 2-year old wood. **(D)** The sum of the annual growth of the lowest six shoots was determined.

To determine whether *MdDAM4* is involved in the regulation of the onset of dormancy, we phenotyped all transgenic and control plants for shoot growth and bud termination. Remarkably, on December 1st, all non-transgenic ‘Gala’ control trees, as well as the *MdDAM4*-overexpressing line M2024, had dropped all leaves. In contrast, all lines for which silencing of *MdDAM4* was assumed were still bearing leaves and had not yet entered dormancy (**Figure 2A**). This indicates that higher *MdDAM4* levels accelerate dormancy onset, whereas reduced *MdDAM4* levels reduce and delay dormancy behavior. Also obvious was the decrease in tree height in line M2024, and an increase in lines with reduced *MdDAM4* levels (**Figures 2A, B**). Lines M2031 to M2050 showed a significant increase in tree height compared with control trees (p = 0.007, T-test). Interestingly, line M2024 was significantly smaller compared with other *MdDAM4* transgenic lines (p = 0.00017, T-test). The same pattern was revealed by measuring the length of annual wood of side shoots (**Figure 2C**). The lengths of annual wood of all side shoots showed a clear trend of decreased annual growth in line M2024 and increased growth in lines with reduced *MdDAM4* levels, which differed not significantly between these lines. The sum of annual wood lengths of the lowest six shoots however differed significantly between transgenic lines M2024 with elevated *MdDAM4* levels and lines with reduced *MdDAM4* levels (M2031, M2047, M2048, M2029, and M2050), which did not differ significantly from each other (p = 0.0023, T-test, **Figure 2D**). Control trees showed an intermediate wood lengths phenotype; however, they did not differ significantly neither from lines with reduced *MdDAM4* levels nor from lines with increased *MdDAM4* levels.

**Figure 3:**
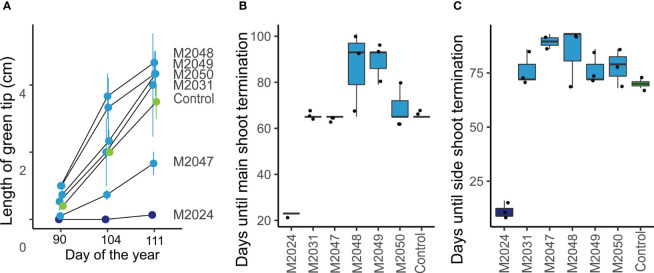
The timing of seasonal growth was altered in *MdDAM4* transgenic trees. **(A)** The length of the green tip outgrowth was measured in all lines at three time points during spring 2021. **(B)** The days until the main shoot had terminated growth were measured from 13/07/2020. **(C)** Similarly, the day was determined until 100% of the side shoot had terminated.

In order to assess, whether the altered annual growth in *MdDAM4* transgenic lines is associated with a change in the length of the growing period, we assessed the timing of beginning and end of growth. In spring, the outgrowth of green leaf tips was significantly less in the *MdDAM4*-overexpressing line M2024 compared with the non-transgenic control (**Figure 3A**). The *MdDAM4-*silenced lines M2031–2050 showed a tendency toward accelerated outgrowth, but this was not significantly different from non-transgenic control. The end of the growth period can be recognized by the termination of bud growth and the development of dormant buds. Therefore, we recorded the dates of main shoot as well as side shoot termination. Line M2024 terminated shoot growth significantly earlier compared with control trees (**Figures 3B, C**). *MdDAM4-*silenced lines again showed a slight tendency of delayed shoot termination, which was not significantly different from control trees. This is true for both the main shoot (**Figure 3B**) and side shoot termination (**Figure 3C**). Whether the altered length of the growing period in *MdDAM4*-transgenic lines is sufficient to explain the difference in annual shoot growth or whether growth rate is also affected by differential *MdDAM4* gene expression requires further exploration. The altered duration of the growth period however is in line with *MdDAM4*’s suggested role in dormancy control.

### 3.3 Effects of altered *MdDAM4* expression on the expression of other *DAM* genes

**Figure 4:**
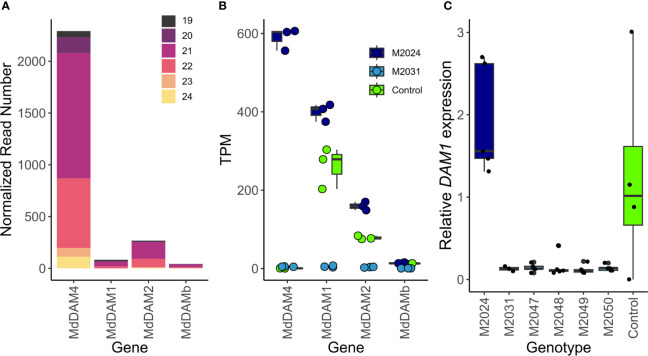
Potential off-targets of transgene-derived siRNAs. **(A)** SiRNAs of 19 to 24 nucleotide in length (represented by different colors) mapped predominantly to *MdDAM4*. A smaller number of siRNAs mapped also to other loci that belong to the *MdSVP* and *MdDAM* gene family, namely, *MdDAM1*, *MdDAM2*, and *MdDAMb*. Reads were normalized to the total number of siRNAs of length 19 to 24. **(B)** RNA-seq data show expression differences of *MdDAM4*, *MdDAM1*, *MdDAM2*, and *MdDAMb* in overexpression line M2014, silenced line M2031, and control. **(C)** Relative *MdDAM1* mRNA expression in control and transgenic apple lines M2024 to M2050, measured by qRT-PCR. Dots of panels **(A, B)** represent measurements of individual replicates, which are summarized by a boxplot showing the median, the 25th percentile, and the 75th percentile.

In order to assess possible off-targets of the *35S:cMdDAM4* transgene, all siRNAs that align with the *MdDAM4* transgenic construct were mapped against the apple transcriptome. As expected, the majority of siRNAs are of 21 nucleotides in length and mapped to the gene *MdDAM4* (**Figure 4A**). Since other *DAM* and *SVP* genes show high sequence similarity to *MdDAM4*, a few siRNAs were observed that map to *MdDAM1*, *MdDAM2*, and *MdDAMb* (**Figure 4A**, **Supplementary Table 2** tab: potential siRNA Off Targets).

Since such off-targets could potentially show reduced expression levels due to siRNAs produced by the transgene and therefore independently of *MdDAM4* activity, we determined the expression levels of *MdDAM4* and of the off-targets *MdDAM1*, *MdDAM2*, and *MdDAMb* from RNA-seq data (see below) (**Figure 4B**). As expected, the overexpression line M2024 showed increased *MdDAM4* levels compared with control and silenced line M2031. At the sampling date, *MdDAM4* was not expressed in control lines. For *MdDAM1* as well as for *MdDAM2*, expression was increased in line M2024 and decreased in line M2031 compared with control. *MdDAMb* showed little expression in all three lines. To confirm our findings, expression of *MdDAM1* was measured also by qRT-PCR in all lines: control, line M2024 and lines M2031 to M2050. Lines M2031–M2050 showed reduced and M2024 showed increased *MdDAM1* expression levels relative to control (**Figure 4C**). These differences however are not significant (p = 0.1 and 0.7, respectively, by t-test). Whether the reduction of *MdDAM1* and *MdDAM2* expression levels are the result of siRNA silencing or whether it is the consequence of reduced *MdDAM4* levels needs to be determined. However, the increase in *MdDAM1* expression in line M2024, which is deplete of siRNAs derived from the transgene, is therefore independent of the siRNAs, which is also true for *MdDAM2*. Further experiments are required in order to determine whether reduced *MdDAM1* expression levels in line M2031 to line M2050 are caused by transgene-derived siRNAs, or whether the absence of the transcription factor *MdDAM4* causes *MdDAM1* expression levels to be lower than in control plants.

### 3.4 Gene expression analysis in transgenic apple lines with overexpressed and silenced *MdDAM4* levels

To gain insight into the molecular mechanisms involved in *MdDAM4-*mediated dormancy control, we performed RNA-seq experiments with the *MdDAM4*-overexpressing line M2024, the silencing line M2031, and non-transgenic control trees.

**Figure 5:**
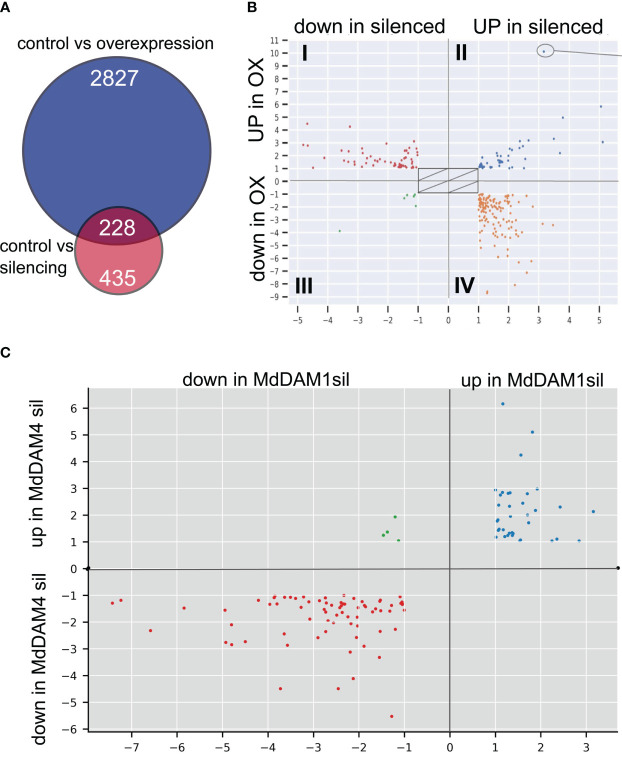
Differentially expressed genes. **(A)** Comparisons of genes differentially expressed between control plants (non-transgenic ‘Gala’) and *MdDAM4*-overexpressing plants of line M2024 (blue circle) as well as control plants and *MdDAM4*-silencing plants of line M2031 (red circle). **(B)** Assignment of DEGs to different groups: group I—DEGs upregulated in the overexpression line M2024 and downregulated in the silenced line M2031. Group II—DEGs upregulated in both genotypes. Group III—DEGs downregulated in in both genotypes. Group IV—DEGs downregulated in the overexpression line M2024 and upregulated in the silenced line M2031. **(C)** The overlap of DEGs of *MdDAM4*-silenced plants with DEGs of *MdDAM1*-silenced plants is mostly in the same direction (blue and red dots). Only four genes show opposite direction in expression.

We found a total of 3.068 differentially expressed genes (DEGs) between non-transgenic control and the *MdDAM4* overexpression line M2024, and 676 DEGs between non-transgenic control and the *MdDAM4* silenced line M2031, with an overlap of 228 DEGs (**Figure 5A**).

The set of 228 genes that were differentially expressed in silenced and overexpression lines were assigned to four groups, depending on line and the direction of the change in expression (**Figure 5B**, **Supplementary Table 2** tab: DEG). Group I consisted of DEGs upregulated in line M2024 and downregulated in line M2031. Genes that were upregulated in both genotypes belonged to group II and genes that were downregulated in both groups belonged to group III. Group IV consisted of genes that were downregulated in line M2024 and upregulated in line M2031. The 59 DEGs that belong to group I are genes, which are potentially induced by *MdDAM4*. Among these are *MdDAM1*, *MdDAM2*, and several genes that are associated with chlorophyll metabolic activity or degradation (**Supplementary Table 2** tab: DEG). The 124 genes of group IV are genes, which are potentially suppressed by *MdDAM4*. Part of this group of genes are three different transcripts that map to homologs of the *cytokinin oxidase 5* (*CKX5)*, five *lipoxygenase (LOX)* homologs (three different *LOX2*s*, LOX3, LOX6*), five transcripts that map to four homologs of the cell wall remodeling *expansins* (*EXP3, EXP8, EXP15, EXP B1*), and several genes involved in cell wall metabolism. Furthermore, the three transcription factors *APETALA 3 (AP3), IBH1-LIKE (IBL1)*, and *EARLY FLOWERING MYB PROTEIN (EFM)* are differentially expressed as well as two WRKY transcription factor genes (*WRKY38, WRKY51*) and the sucrose transporter *SUC2.* Genes of group II and IV are of little interest, since the cause for the differential expression likely is independent of *MdDAM4.* Common to both lines and different to controls are few steps in generating transgenic trees.

Gene ontology analysis that were performed for all DEGs in the *MdDAM4*-overexpressing line M2024 indicate cytoskeleton and components of photosynthesis to be affected by overexpression of *MdDAM4*. No significant GO enrichments were found in DEGs in silenced line M2031, nor in DEGs common to both silenced and overexpressing *MdDAM4* lines.

### 3.5 Genes differentially expressed in both *MdDAM1-*silenced and *MdDAM4*-silenced lines

Silencing of *MdDAM4* and *MdDAM1* affects common as well as unique targets. Here, a comparison was done between differentially expressed transcripts observed in our previous work in *MdDAM1*-silenced plants (Moser et al., 2020) and differentially expressed transcripts of *MdDAM4*-silenced plants. Three types of behaviors were observed: 36 transcripts were upregulated in *MdDAM1*-silenced plants and *MdDAM4*-silenced plants as well; 72 transcripts showed downregulation in both cases; and 4 transcripts were downregulated in the *MdDAM1* silenced line but upregulated in the *MdDAM4*-silenced line (**Figure 5C**, **Supplementary Table 2** tab: MdDAM4_vs_MdDAM1_DEG). No transcripts were found that were down-regulated in the *MdDAM4*-silenced line and up-regulated in the *MdDAM1*-silenced line. Among the genes that are downregulated in both *MdDAM1* and *MdDAM4* silenced lines, are *MdDAM2*, *MdDAM4*, *MdDAMb*, and *MdSVPa*. It is unclear whether the downregulation of these genes is a side effect of siRNA-mediated gene silencing of very close homologs or whether there is transcriptional regulation involved.

## 4 Discussion

The results of this study confirm *MdDAM4*’s essential role in the control of winter dormancy in apple. Transgenic apple trees with impaired *MdDAM4* function clearly show an altered onset of growth cessation that accompanies the onset of winter dormancy. The overexpression of *MdDAM4* with the 35S promoter resulted in both a line that overexpressed the gene of interest (line M2024), as well as five lines (M2031 and M2047–M2050) in which *MdDAM4* was suppressed. This phenomenon can be explained for four transgenic lines (M2031, M2047, M2029, and M2050) by siRNAs that derive from the transgene and map to the coding region as well as the tOCS terminator. Transgene silencing is a phenomenon that has been observed frequently in transgenic lines, especially in combination with the 35S promoter (De Paoli et al., 2009; Yao et al., 2018; Moser et al., 2020). Additional to the overexpression via the 35S promoter, multiple insertions of the T-DNA in the genome can also cause gene silencing (Schubert et al., 2004; Moser et al., 2020). The *MdDAM4* transgenic lines of this study also show silencing in lines with multiple insertions, with one exception, and no silencing in line M2024 with a single T-DNA insertion. This indicates that multiple insertions cause gene silencing in these lines. Despite multiple T-DNA insertions in line M2048, this line shows no siRNAs that map to *MdDAM4*. Phenotypically, this line resamples lines with silenced *MdDAM4.* Therefore, we hypothesize that aberrant transcripts longer than 24 bp may lead to gene silencing in this line. However, final evidence for this hypothesis is still missing. Further research is still needed.

Phenotypically, transgenic trees with altered *MdDAM4* expression levels stand out by their altered phenology. Apple trees with elevated *MdDAM4* levels (M2024) stopped their growth period and dropped leaves earlier than the non-transgenic control plants and trees with reduced *MdDAM4* levels (M2031, M2047, M2048, M2029, and M2050) showed a prolonged growth period and did not drop their leaves during fall. Furthermore, these transgenic trees showed increased or reduced annual growth depending on *MdDAM4* levels. Overexpression leads to reduced growth, and silencing leads to an increase of growth. A change in annual growth can be caused either by a change in growth rate or by changing the length of the growing period. Although we did not investigate growth rate in these lines, the presented results on *MdDAM4* transgenic lines clearly indicate that *MdDAM4* is involved in controlling shoot termination and the onset of winter dormancy in the fall. Therefore, our results suggest that the transcription factor *MdDAM4* is involved in controlling the timing of growth cessation and the end of the growing season. To gain further knowledge on *MdDAM4* is of high interest, as the tight control of the growing season and its alignment with environmental growth conditions is key to annual growth and reproductive success (Myneni et al., 1997; Chuine and Beaubien, 2002; Chuine et al., 2010; Vitasse et al., 2018). Further experiments are required to determine which molecular factors and which environmental cues control *MdDAM4* expression and activity.

In this study, differential gene expression was surveyed as downstream effects of *MdDAM4*. The overexpression line M2024 differed phenotypically more significantly from non-transgenic control trees, which was not always the case for silencing line M2031. Therefore, it is not surprising that a 4.5 times larger number of differentially expressed genes was found in *MdDAM4*-overexpressing samples compared with samples with *MdDAM4* silencing. Among the genes that were potentially activated or suppressed by *MdDAM4* were several genes that can be linked to leaf senescence. Among these were several genes involved in chlorophyll metabolic activity, as well as the sucrose transporter SUC2, which in *A. thaliana* is essential for phloem loading and long-distance transport (Durand et al., 2018). Additionally, several homologs of the *cytokinin oxidase 5* (*CKX5)* were found to be potentially suppressed by *MdDAM4*. In *A. thaliana*, *CKX5* is expressed in meristems and degrades active cytokinin. Mutants lacking functional *CKX3* and *CKX5* show floral meristems of increased size and at the same time with retarded differentiation (Bartrina et al., 2011). In apple, the progression of differentiation of floral meristems stops during winter months (Zeller, 1958). It would be interesting to investigate whether *MdDAM4* controls the retardation of floral meristem differentiation during winter and whether it does so via cytokinin and *CKX5*. The cytokinin B-type response regulators have been found in previous studies to play a role in winter dormancy, and *MdBRR9* showed a correlation of expression with ambient temperature (Cattani et al., 2020; Lempe et al., 2022). Cytokinins also play a role in leaf senescence—high levels of cytokinin delay leaf senescence (Sarwat et al., 2013). Additionally to *CKX5*, five *LOX* homologs were found in this study to be potentially suppressed by *MdDAM4*. *LOX* genes are enzymes that catalyze the first step in the biosynthesis of biologically active oxylipins, of which jasmonate is the best known (Howe and Schilmiller, 2002). Apart from stress response, jasmonic acid (JA) also plays a role in the process of leaf senescence (He et al., 2002; Schommer et al., 2008; Sarwat et al., 2013; Springer et al., 2016). Furthermore, five expansins, which are able to remodel cell wall stability, were shown to be differentially expressed (Cosgrove, 2000). The processes of leaf senescence, jasmonic acid biosynthesis, and cell wall remodeling all occur during the activation of the abscission zone (AZ) (Kim et al., 2019). The AZ is a specialized tissue that is created during tissue development and stays in an undifferentiated state throughout organ development. Upon perception of abscission signals, cells in the AZ differentiate, cell walls are remodeled, programmed cell death occurs, the tissue can separate, and a new protective layer is formed. Leaf senescence as well as the formation of the AZ could potentially be involved in the process of nutrient reabsorption and leaf drop in fall. Transgenic trees overexpressing *MdDAM4* drop their leaves earlier compared with non-transgenic control; when *MdDAM4* is silenced, leaf drop is much delayed. In order to draw a final conclusion on *MdDAM4*’s role in the process of leaf senescence, more work is required. Especially, the spatial resolution of the differentially expressed genes needs to be resolved. The direction of expression does not support a role of *MdDAM4* in promoting leaf senescence and leaf drop, when considering *CKX5, LOX* and *expansins—*they were upregulated in silenced and downregulated in overexpression lines and are therefore potentially suppressed by *MdDAM4*. Gene expression however was measured in bud tissue and not in older leaves. It would be interesting to test further whether *MdDAM4* is expressed in older leaves at all and whether it directly promotes leaf senescence and AZ formation in older leaves, or whether it is expressed in floral bud tissue only, where it could potentially protect bud tissue from senescing during fall.

**Figure 6:**
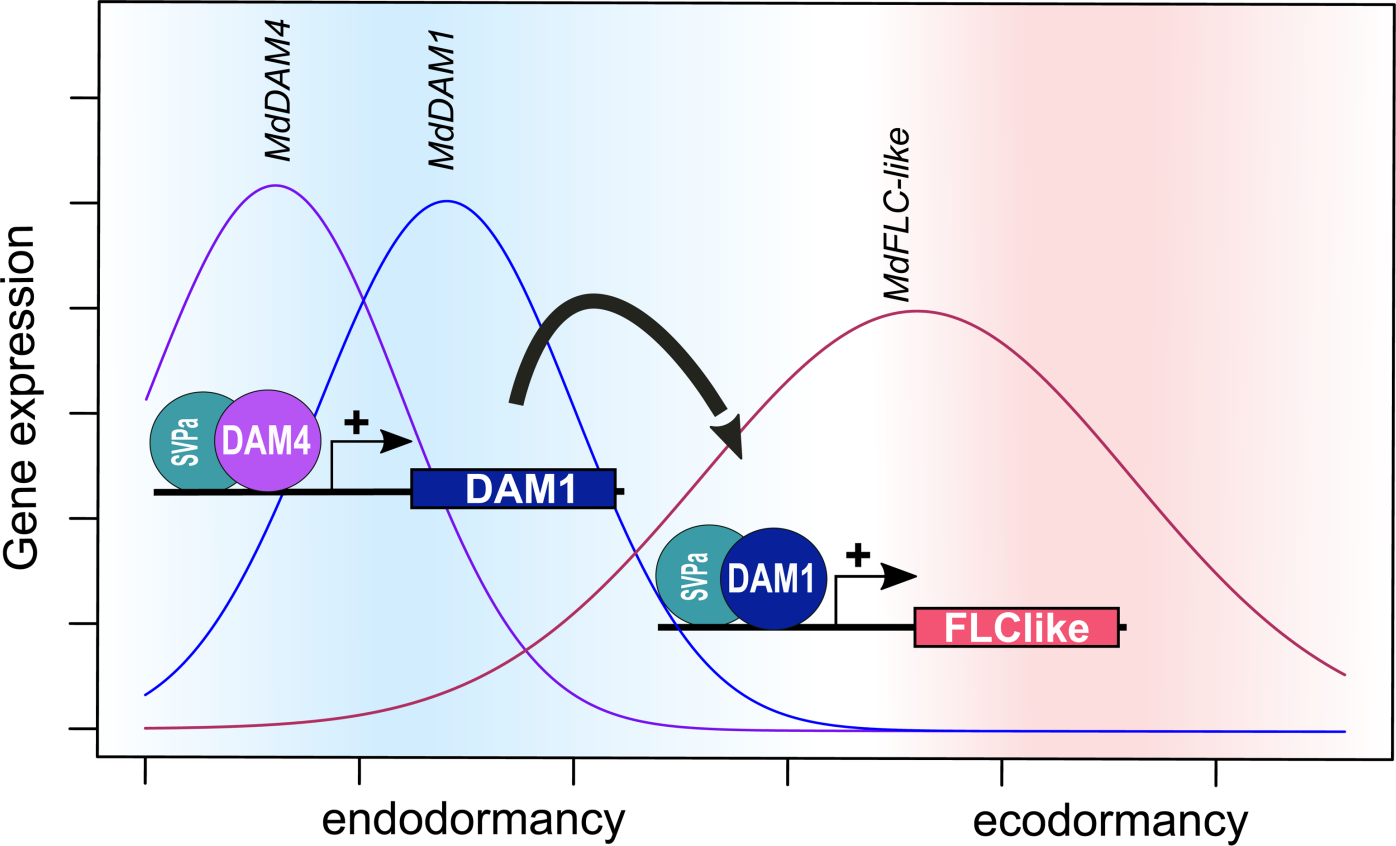
Tentative model of the temporal gene expression of *MdDAM4, MdDAM1*, and *MdFLC-like* during bud dormancy in apple. Based on the expression pattern described in Lempe et al. (2022), the results of DAP-seq obtained by Falavigna et al. (2021), the RNA-seq experiments done by Moser et al., 2020, and results of the present study, we propose the following model: the increase in expression of *MdDAM4* during early endodormancy allows the formation of a heteromeric protein complex containing *MdDAM4* and *MdSVPa* proteins. This complex binds to the promoter region of *MdDAM1* and induces its expression. Subsequently, the heteromeric protein complex of *MdDAM1* and *MdSVPa* is formed and binds to the promoter region of *MdFLC-like*.

Phenotypically, the silencing of either *MdDAM1* or *MdDAM4* had very similar effects (Moser et al., 2020). It is an open question whether *MdDAM4* can influence the activity of *MdDAM1* or vice versa. *MdDAM1* and *MdDAM4* both showed a more or less strong peak of expression during endodormancy; however, expression patterns varied between cultivar and climatic condition (Moser et al., 2020; Falavigna et al., 2021; Lempe et al., 2022). In South Tirol, the expression patterns of *MdDAM1* and *MdDAM4* were very similarly in ‘Golden Delicious’; in contrast, the low chill cultivars ‘Dorsett Golden’ and ‘Anna’ show lower levels of *MdDAM1* expression and no *MdDAM4* expression (Moser et al., 2020). In Southern France, ‘Royal Gala’ showed a broad expression curve for *MdDAM1* and *MdDAM4* with decreasing levels after the endo- to ecodormancy transition (Falavigna et al., 2021). In eastern Germany, *MdDAM4* expression peaked slightly earlier than *MdDAM1* in both ‘Gala’ and ‘Pinova’, although the expression peak of *MdDAM4* was lower in ‘Gala’ compared with ‘Pinova’ (Lempe et al., 2022). Since there is high sequence similarity between MADS-box transcription factors *MdDAM1* and *MdDAM4*, the siRNAs that were generated in *MdDAM4* transgenic lines could target also *MdDAM1*, although to a much lesser extent. The same is true for the transgenic *MdDAM1* lines (Moser et al., 2020). Therefore, it was not possible to disentangle whether a reduction of expression was due to gene silencing or due to a reduction in transcriptional activation. However, the *MdDAM4*-overexpressing line M2024 showed increased levels of *MdDAM1*, which indicates that *MdDAM4* could induce *MdDAM1* expression. The transcriptional activation of *MdDAM1* by *MdDAM4* is in line with the findings of Falavigna et al. (2021), a study that provides experimental evidence of the binding of the MdDAM4–MdSVPa transcription factor complex to the *MdDAM1* promoter region. Therefore, based on this evidence, we conclude a temporal sequence where *MdDAM4* induces *MdDAM1* and *MdDAM1* subsequently induces the expression of *MdFLC-like* (**Figure 6**). In the lines M2024 and M2031, *MdDAM2* shows similar expression patterns to *MdDAM1* and could therefore potentially also be induced by *MdDAM4*. However, during the course of winter dormancy, *MdDAM2* is expressed earlier than *MdDAM4*, before endodormancy (Falavigna et al., 2021; Lempe et al., 2022). The role *MdDAM2* plays during dormancy needs to be determined. It also differs from *MdDAM1*, *MdDAM4*, *MdDAMb*, and *MdFLC*-like in its dimerization ability. *MdDAM2* does not interact with MdSVP1 or MdSVPb (Falavigna et al., 2021). Duplication and sequence divergence of members of the MADS-box transcription factor family have played an important role in the evolution of land plants (Kaufmann et al., 2005; Qiu and Köhler, 2022). Especially features like the combinatorial formation of heterodimers enabled additional fine-tuning of transcriptional control. Also in Rosaceae, *SVP* and *DAM* genes have undergone gene expansions by whole-genome as well as tandem gene duplication and subsequent functional diversification (Liu et al., 2020; Quesada-Traver et al., 2022). Also, the formation of heterodimers of *SVP* and *DAM* genes has been observed, which potentially increases the complexity of transcriptional control (Falavigna et al., 2021). Similar to work on *MdDAM1*, the results of this study imply *MdDAM4* in the control of the onset of endodormancy (Moser et al., 2020). How environmental factors shape the temporal expression space of *MdDAM1* and *MdDAM4*, where these transcription factors are expressed locally, and how these two MADS-box genes have functionally diverged are not yet understood.

## Data availability statement

The datasets presented in this study can be found in online repositories. The names of repostheitory/repositories and accession number(s) can be found here: https://www.ncbi.nlm.nih.gov/genbank/, OR394976 https://www.ncbi.nlm.nih.gov/, BioProject ID PRJNA1101740.

## Author contributions

JL: Conceptualization, Data curation, Formal analysis, Writing – original draft, Writing – review & editing, Visualization. MM: Conceptualization, Data curation, Formal analysis, Writing – original draft, Writing – review & editing. EA: Data curation, Writing – original draft, Writing – review & editing. AS-A: Conceptualization, Writing – original draft, Writing – review & editing, Funding acquisition. HF: Conceptualization, Writing – original draft, Writing – review & editing, Funding acquisition, Project administration, Supervision.

## References

[B1] Atkinson C. J. Brennan R. M. Jones H. G. (2013). Declining chilling and its impact on temperate perennial crops. Environ. Exp. Bot. 91, 48–62. doi: 10.1016/j.envexpbot.2013.02.004

[B2] Bartrina I. Otto E. Strnad M. Werner T. Schmülling T. (2011). Cytokinin regulates the activity of reproductive meristems, flower organ size, ovule formation, and thus seed yield in. Plant Cell 23, 69–80. doi: 10.1105/tpc.110.079079 21224426 PMC3051259

[B3] Bielenberg D. G. Wang Y. Li Z. G. Zhebentyayeva T. Fan S. H. Reighard G. L.. (2008). Sequencing and annotation of the evergrowing locus in peach [*Prunus persica* (L.) Batsch] reveals a cluster of six MADS-box transcription factors as candidate genes for regulation of terminal bud formation. Tree Genet. Genomes 4, 495–507. doi: 10.1007/s11295-007-0126-9

[B4] Bolger A. M. Lohse M. Usadel B. (2014). Trimmomatic: a flexible trimmer for Illumina sequence data. Bioinformatics 30 (15), 2114–2120. doi: 10.1093/bioinformatics/btu170 24695404 PMC4103590

[B5] Cattani A. M. da Silveira Falavigna V. Silveira C. P. Buffon V. Dos Santos Maraschin F. Pasquali G.. (2020). Type-B cytokinin response regulators link hormonal stimuli and molecular responses during the transition from endo- to ecodormancy in apple buds. Plant Cell Rep. 39, 1687–1703. doi: 10.1007/s00299-020-02595-z 32959122

[B6] Chuine I. Beaubien E. G. (2002). Phenology is a major determinant of tree species range. Ecol. Lett. 5, 316–316. doi: 10.1046/j.1461-0248.2001.00261.x

[B7] Chuine I. Morin X. Bugmann H. (2010). Warming, photoperiods, and tree phenology. Science 329, 277–278. doi: 10.1126/science.329.5989.277-e 20647448

[B8] Cooke J. E. K. Eriksson M. E. Junttila O. (2012). The dynamic nature of bud dormancy in trees: environmental control and molecular mechanisms. Plant Cell Environ. 35, 1707–1728. doi: 10.1111/j.1365-3040.2012.02552.x 22670814

[B9] Cosgrove D. J. (2000). Loosening of plant cell walls by expansins. Nature 407, 321–326. doi: 10.1038/35030000 11014181

[B10] De Paoli E. Dorantes-Acosta A. Zhai J. X. Accerbi M. Jeong D. H. Park S.. (2009). Distinct extremely abundant siRNAs associated with cosuppression in petunia. Rna 15, 1965–1970. doi: 10.1261/rna.1706109 19776157 PMC2764480

[B11] Durand M. Mainson D. Porcheron B. Maurousset L. Lemoine R. Pourtau N. (2018). Carbon source-sink relationship in *A.thaliana*: the role of sucrose transporters. Planta 247, 587–611. doi: 10.1007/s00425-017-2807-4 29138971 PMC5809531

[B12] Falavigna V. D. S. Severing E. Lai X. Estevan J. Farrera I. Hugouvieux V.. (2021). Unraveling the role of MADS transcription factor complexes in apple tree dormancy. New Phytol. 232 (5), 2071–2088. doi: 10.1111/nph.17710 34480759 PMC9292984

[B13] Fernandez E. Mojahid H. Fadon E. Rodrigo J. Ruiz D. Egea J. A.. (2023). Climate change impacts on winter chill in Mediterranean temperate fruit orchards. Regional Environ. Change 23 (1), 1–18. doi: 10.1007/s10113-022-02006-x

[B14] Flachowsky H. Peil A. Sopanen T. Elo A. Hanke V. (2007). Overexpression of *BpMADS4* from silver birch (*Betula pendula* Roth.) induces early-flowering in apple (*Malus x domestica* Borkh.). Plant Breed. 126, 137–145. doi: 10.1111/j.1439-0523.2007.01344.x

[B15] He Y. H. Fukushige H. Hildebrand D. F. Gan S. S. (2002). Evidence supporting a role of jasmonic acid in Arabidopsis leaf senescence. Plant Physiol. 128, 876–884. doi: 10.1104/pp.010843 11891244 PMC152201

[B16] Heide O. M. (2008). Interaction of photoperiod and temperature in the control of growth and dormancy of Prunus species. Scientia Hortic. 115, 309–314. doi: 10.1016/j.scienta.2007.10.005

[B17] Heide O. M. Prestrud A. K. (2005). Low temperature, but not photoperiod, controls growth cessation and dormancy induction and release in apple and pea. Tree Physiol. 25, 109–114. doi: 10.1093/treephys/25.1.109 15519992

[B18] Horvath D. P. Sung S. Kim D. Chao W. Anderson J. (2010). Characterization, expression and function of DORMANCY ASSOCIATED MADS-BOX genes from leafy spurge. Plant Mol. Biol. 73, 169–179. doi: 10.1007/s11103-009-9596-5 20066557

[B19] Howe G. A. Schilmiller A. L. (2002). Oxylipin metabolism in response to stress. Curr. Opin. Plant Biol. 5, 230–236. doi: 10.1016/S1369-5266(02)00250-9 11960741

[B20] Kassambara A. (2023). ggpubr: ‘ggplot2’ Based publication ready plots. Available online at: https://rpkgs.datanovia.com/ggpubr/.

[B21] Kaufmann K. Melzer R. Theissen G. (2005). MIKC-type MADS-domain proteins: structural modularity, protein interactions and network evolution in land plants. Gene 347, 183–198. doi: 10.1016/j.gene.2004.12.014 15777618

[B22] Kim J. Chun J. P. Tucker M. L. (2019). Transcriptional regulation of abscission zones. Plants-Basel 8. doi: 10.3390/plants8060154 PMC6631628 31174352

[B23] Kumar G. Arya P. Gupta K. Randhawa V. Acharya V. Singh A. K. (2016). Comparative phylogenetic analysis and transcriptional profiling of MADS-box gene family identified *DAM* and *FLC-like* genes in apple (*Malus* x *domestica*). Sci. Rep. 6, 20695. doi: 10.1038/srep20695 26856238 PMC4746589

[B24] Lang G. A. (1987). Dormancy: A new universal terminology. Hortic. Sci. 22, 817–820. doi: 10.21273/HORTSCI.22.5.817

[B25] Legave J. M. Blanke M. Christen D. Giovannini D. Mathieu V. Oger R. (2013). A comprehensive overview of the spatial and temporal variability of apple bud dormancy release and blooming phenology in Western Europe. Int. J. Biometeorol. 57, 317–331. doi: 10.1007/s00484-012-0551-9 22610120

[B26] Lempe J. Peil A. Flachowsky H. (2022). Time-resolved analysis of candidate gene expression and ambient temperature during bud dormancy in apple. Front. Plant Sci. 12. doi: 10.3389/fpls.2021.803341 PMC8802299 35111181

[B27] Liu J. Ren M. Chen H. Wu S. Yan H. Jalal A.. (2020). Evolution of *SHORT VEGETATIVE PHASE (SVP)* genes in Rosaceae: Implications of lineage-specific gene duplication events and function diversifications with respect to their roles in processes other than bud dormancy. Plant Genome 13, e20053. doi: 10.1002/tpg2.20053 33217197 PMC12807081

[B28] Love M. I. Huber W. Anders S. (2014). Moderated estimation of fold change and dispersion for RNA-seq data with DESeq2. Genome Biology 15 (12). doi: 10.1186/s13059-014-0550-8 PMC4302049 25516281

[B29] Mimida N. Saito T. Moriguchi T. Suzuki A. Komori S. Wada M. (2015). Expression of *DORMANCY-ASSOCIATED MADS-BOX (DAM)*-like genes in apple. Biol. Plantarum 59, 237–244. doi: 10.1007/s10535-015-0503-4

[B30] Moser M. Asquini E. Miolli G. V. Weigl K. Hanke M. V. Flachowsky H.. (2020). The MADS-box gene *MdDAM1* controls growth cessation and bud dormancy in apple. Front. Plant Sci. 11. doi: 10.3389/fpls.2020.01003 PMC7358357 32733512

[B31] Myneni R. B. Keeling C. D. Tucker C. J. Asrar G. Nemani R. R. (1997). Increased plant growth in the northern high latitudes from 1981 to 1991. Nature 386, 698–702. doi: 10.1038/386698a0

[B32] Pfleiderer P. Menke I. Schleussner C. F. (2019). Increasing risks of apple tree frost damage under climate change. Climatic Change 157, 515–525. doi: 10.1007/s10584-019-02570-y

[B33] Porto D. D. Bruneau M. Perini P. Anzanello R. Renou J. P. dos Santos H. P.. (2015). Transcription profiling of the chilling requirement for bud break in apples: a putative role for FLC-like genes. J. Exp. Bot. 66, 2659–2672. doi: 10.1093/jxb/erv061 25750421

[B34] Qiu Y. C. Köhler C. (2022). Endosperm evolution by duplicated and neofunctionalized type I MADS-box transcription factors. Mol. Biol. Evol. 39 (1), msab355. doi: 10.1093/molbev/msab355 34897514 PMC8788222

[B35] Quesada-Traver C. Lloret A. Carretero-Paulet L. Badenes M. L. Ríos G. (2022). Evolutionary origin and functional specialization of Dormancy-Associated MADS box (DAM) proteins in perennial crops. BMC Plant Biol. 22 (1), 473. doi: 10.1186/s12870-022-03856-7 36199018 PMC9533583

[B36] Rodriguez J. Sherman W. B. Scorza R. Wisniewski M. Okie W. R. (1994). Evergreen peach, its inheritance and dormant behavior. J. Am. Soc. Hortic. Sci. 119, 789–792. doi: 10.21273/JASHS.119.4.789

[B37] Saito T. Bai S. L. Ito A. Sakamoto D. Saito T. Ubi B. E.. (2013). Expression and genomic structure of the genes *MADS13* in Japanese pears (*Pyrus pyrifolia* Nakai) that differ in their chilling requirement for endodormancy release. Tree Physiol. 33, 654–667. doi: 10.1093/treephys/tpt037 23761324

[B38] Sarwat M. Naqvi A. R. Ahmad P. Ashraf M. Akram N. A. (2013). Phytohormones and microRNAs as sensors and regulators of leaf senescence: Assigning macro roles to small molecules. Biotechnol. Adv. 31, 1153–1171. doi: 10.1016/j.biotechadv.2013.02.003 23453916

[B39] Sasaki R. Yamane H. Ooka T. Jotatsu H. Kitamura Y. Akagi T.. (2011). Functional and expressional analyses of *pmDAM* genes associated with endodormancy in Japanese apricot. Plant Physiol. 157, 485–497. doi: 10.1104/pp.111.181982 21795580 PMC3165894

[B40] Schommer C. Palatnik J. F. Aggarwal P. Chételat A. Cubas P. Farmer E. E.. (2008). Control of jasmonate biosynthesis and senescence by miR319 targets. PloS Biol. 6, 1991–2001. doi: 10.1371/journal.pbio.0060230 PMC2553836 18816164

[B41] Schubert D. Lechtenberg B. Forsbach A. Gils M. Bahadur S. Schmidt R. (2004). Silencing in Arabidopsis T-DNA transformants: The predominant role of a gene-specific RNA sensing mechanism versus position effects. Plant Cell 16, 2561–2572. doi: 10.1105/tpc.104.024547 15367719 PMC520955

[B42] Singh R. K. Maurya J. P. Azeez A. Miskolczi P. Tylewicz S. Stojkovic K.. (2018). A genetic network mediating the control of bud break in hybrid aspen. Nat. Commun. 9 (1), 4173. doi: 10.1038/s41467-018-06696-y 30301891 PMC6177393

[B43] Springer A. Kang C. Rustgi S. von Wettstein D. Reinbothe C. Pollmann S.. (2016). Programmed chloroplast destruction during leaf senescence involves 13-lipoxygenase (13-LOX). Proc. Natl. Acad. Sci. United States America 113, 3383–3388. doi: 10.1073/pnas.1525747113 PMC4812756 26969728

[B44] Takeuchi T. Matsushita M. C. Nishiyama S. Yamane H. Banno K. Tao R. (2018). RNA-sequencing analysis identifies genes associated with chilling-mediated endodormancy release in apple. J. Am. Soc. Hortic. Sci. 143, 194–206. doi: 10.21273/JASHS04345-18

[B45] Tian T. Liu Y. Yan H. You Q. Yi X. Du Z.. (2017). agriGO v2.0: a GO analysis toolkit for the agricultural community 2017 update. Nucleic Acids Res. 45, W122–W129. doi: 10.1093/nar/gkx382 28472432 PMC5793732

[B46] Unterberger C. Brunner L. Nabernegg S. Steininger K. W. Steiner A. K. Stabentheiner E.. (2018). Spring frost risk for regional apple production under a warmer climate. PloS One 13 (7), e0200201. doi: 10.1371/journal.pone.0200201 30044808 PMC6059414

[B47] Vergara R. Noriega X. Perez F. J. (2021). VvDAM-SVPs genes are regulated by FLOWERING LOCUS T (VvFT) and not by ABA/low temperature-induced VvCBFs transcription factors in grapevine buds. Planta 253, 31. doi: 10.1007/s00425-020-03561-5 33438039

[B48] Vitasse Y. Baumgarten F. Zohner C. M. Rutishauser T. Pietragalla B. Gehrig R.. (2022). The great acceleration of plant phenological shifts. Nat. Climate Change 12, 300–302. doi: 10.1038/s41558-022-01283-y

[B49] Vitasse Y. Signarbieux C. Fu Y. S. H. (2018). Global warming leads to more uniform spring phenology across elevations. Proc. Natl. Acad. Sci. United States America 115, 1004–1008. doi: 10.1073/pnas.1717342115 PMC5798366 29279381

[B50] Wickham H. (2016). ggplot2: elegant graphics for data analysis (New York: Springer-Verlag). doi: 10.1007/978-3-319-24277-4

[B51] Wisniewski M. Norelli J. Artlip T. (2015). Overexpression of a peach *CBF* gene in apple: a model for understanding the integration of growth, dormancy, and cold hardiness in woody plants. Front. Plant Sci. 6. doi: 10.3389/fpls.2015.00085 PMC4343015 25774159

[B52] Wu R. Cooney J. Tomes S. Rebstock R. Karunairetnam S. Allan A. C.. (2021). RNAi-mediated repression of dormancy-related genes results in evergrowing apple trees. Tree Physiol. 41 (8), 1510–1523. doi: 10.1093/treephys/tpab007 33564851

[B53] Wu R. Tomes S. Karunairetnam S. Tustin S. D. Hellens R. P. Allan A. C.. (2017). SVP-like MADS box genes control dormancy and budbreak in apple. Front. Plant Sci. 8. doi: 10.3389/fpls.2017.00477 PMC5378812 28421103

[B54] Wu R. M. Walton E. F. Richardson A. C. Wood M. Hellens R. P. Varkonyi-Gasic E. (2012). Conservation and divergence of four kiwifruit *SVP*-like MADS-box genes suggest distinct roles in kiwifruit bud dormancy and flowering. J. Exp. Bot. 63, 797–807. doi: 10.1093/jxb/err304 22071267 PMC3254681

[B55] Yao J. L. Xu J. Tomes S. Cui W. Luo Z. W. Deng C.. (2018). Ectopic expression of the *PISTILLATA* homologous *MdPI* inhibits fruit tissue growth and changes fruit shape in apple. Plant Direct 2 (4), e00051. doi: 10.1002/pld3.51 31245717 PMC6508508

[B56] Zeller O. (1958). Über die Jahresrhythmik in der Entwicklung der Blütenknospen einiger Obstsorten. Gartenbauwissenschaften 23, 167–181.

