# Supplementary figures and images for: Functional evidence on the involvement of the MADS-box gene MdDAM4 in bud dormancy regulation in apple
Source: Front Plant Sci. 2024 Jul 15;15:1433865. doi: 10.3389/fpls.2024.1433865 (PMC11284153; doi:10.3389/fpls.2024.1433865)

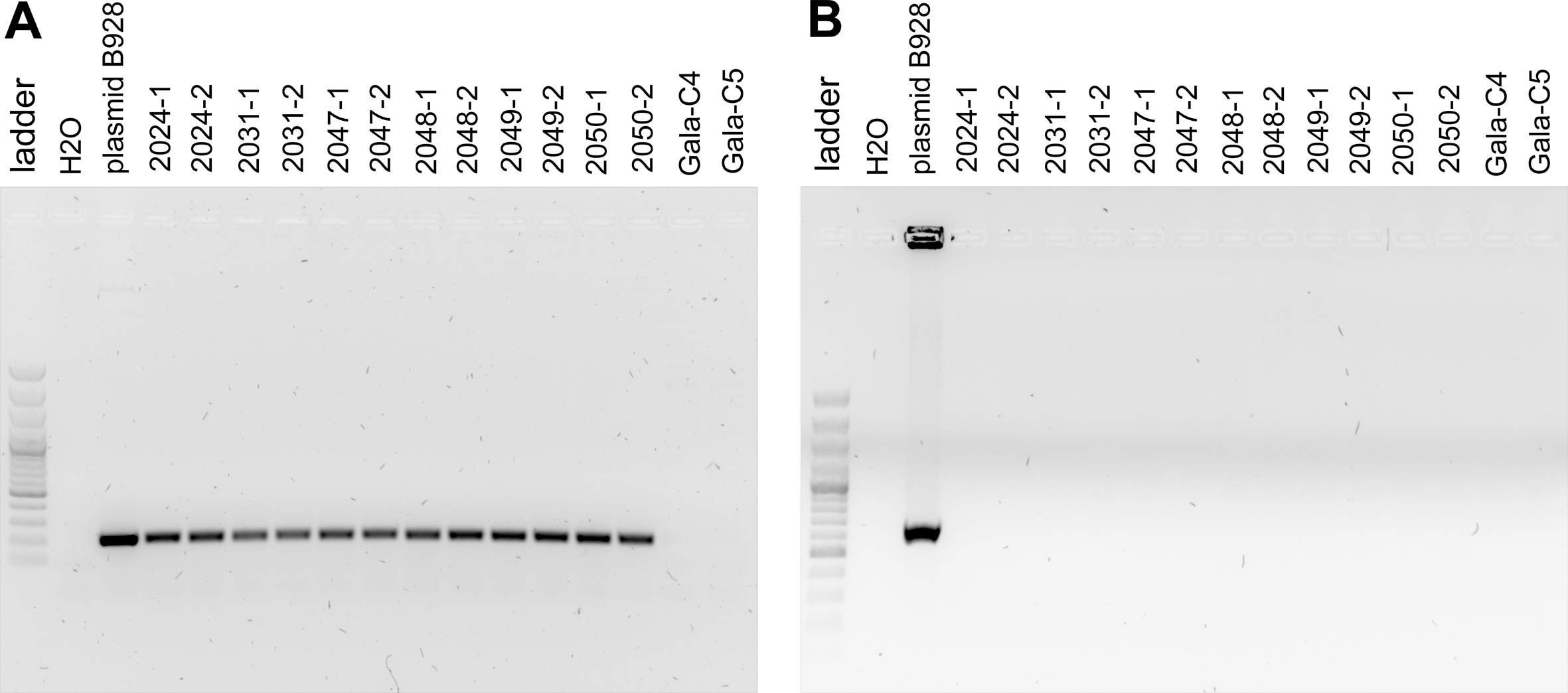

Supplement: Supplementary file 1 [file Image_1.tif]
